# Validation of New Allele-Specific Real-Time PCR System for Thiopurine Methyltransferase Genotyping in Korean Population

**DOI:** 10.1155/2013/305704

**Published:** 2013-02-28

**Authors:** Sollip Kim, Hye Won Lee, Woochang Lee, Sail Chun, Won-Ki Min

**Affiliations:** ^1^Department of Laboratory Medicine, Ilsan Paik Hospital, Inje University College of Medicine, Joowha-ro 170, Ilsanseo-gu, Goyang, Gyeonggi, Republic of Korea; ^2^Department of Laboratory Medicine, Asan Medical Center, University of Ulsan College of Medicine, 88, Olympic-ro 43-gil, Songpa-gu, Seoul, Republic of Korea

## Abstract

*Introduction*. Thiopurine drugs are metabolized via S-methylation and catalyzed by thiopurine S-methyltransferase (TPMT). Patients with very low TPMT activity are at high risk of fatal bone marrow toxicity when standard doses of thiopurine drugs are administered. *TPMT* genotyping can predict TPMT activity and is not affected by transfusion or red blood cell defects. Here, we report a new allele-specific real-time polymerase chain reaction (PCR) system for thiopurine methyltransferase genotyping that is validated in Korean population. *Materials and Methods*. Three major *TPMT* single-nucleotide polymorphisms (*TPMT*∗*2*, ∗*3B*, and ∗*3C*) were genotyped using real-time PCR with the allele-specific primers and probes. Internal positive controls were included in each well, and an automatic interpretative algorithm was applied. This system was validated using 244 clinical samples and 2 commercial DNA samples that had been previously genotyped using PCR-direct sequencing. *Results*. All of the obtained results are concordant with those of the reference method. All of the internal positive control reactions were successful. The allele frequency of *TPMT*∗*3C* was 2.05% (10 of 488 alleles). All of the patients with variant alleles were heterozygotes, and no homozygotes were detected. No *TPMT*∗*2*, ∗*3A*, or ∗*3B* alleles were observed in this Korean population. *Conclusion*. This rapid, accurate, and user-friendly genotyping system can be readily used to improve the efficacy and safety of thiopurine treatments in clinical practice.

## 1. Introduction

Thiopurine drugs, such as azathioprine, 6-mercaptopurine, and 6-thioguanine, are used to treat patients with leukemia, inflammatory bowel disease, rheumatic disease, and those who have received an organ transplant [1]. Approximately 30–40% of inflammatory bowel disease patients fail to benefit from this treatment [2]. The crucial factor that explains the interindividual differences, in terms of therapeutic efficacy and adverse reactions, is the variable activity of the thiopurine methyltransferase (TPMT) enzyme, which is involved in the metabolic pathways of these drugs [2]. Approximately 90% of individuals have normal activity, 10% have intermediate activity, and 0.3% have low or undetectable activity [1]. Patients with decreased TPMT activity might experience hematopoietic toxicity, such as myelosuppression, if they are treated with standard thiopurine doses [3, 4]. Dosage reduction can minimize toxicity in these patients [5].

Methods that measure the TPMT activity of red blood cells (RBCs) are available, but these results may be falsely elevated by recent blood transfusions or falsely lowered by RBC aging [6, 7]. On the other hand, *TPMT* genotyping can predict TPMT activity and offers an advantage over phenotypic methods in the aforementioned situations [1, 6, 8]. Compared with wild-type (TPMT *1) homozygotes, individuals with 2 variant alleles demonstrate low or undetectable TPMT activity, while those with one variant allele demonstrate intermediate TPMT activity [1]. 

Although polymerase-chain-reaction-(PCR-) direct sequencing is considered the gold standard, both the test procedures and the interpretation of the results are time-consuming and labor-intensive. Moreover, four variant alleles together account for over 95% of the reduced TPMT activity in Caucasian subjects [9]: TPMT *2 (rs1800462, c.238G > C, and p.Ala80Pro); *3B (rs1800460, c.460G > A, and p.Ala154Thr); *3C (rs1142345, c.719A > G, and p.Tyr240Cys); *3A (c.460G > A and c.719A > G). The most prevalent variant alleles in the Caucasian population is TPMT *3A (10% of this population carries a nonfunctional allele), while TPMT *3C predominates in the East Asian population (4.7% of this population) [2]. 

Therefore, genotyping only the major target regions could be sufficient for preassessment of patients prior to commencing thiopurine drugs. In addition, a faster, high-throughput method is needed for applications in the clinical laboratory. Allele-specific real-time PCR may be a good approach. In this paper, we describe how we developed and validated a new allele-specific real-time PCR system with an automatic interpretative function that can be used to detect* TPMT *genetic polymorphisms for the dose adjustment of thiopurine drugs.

## 2. Materials and Methods

### 2.1. Development of the Allele-Specific Real-Time PCR System

Our allele-specific, real-time PCR system was developed based on the use of allele-specific primers and 5′ nuclease probes. Two types of primers were designed in order to specifically detect the desired genotypes: one specific for the wild-type allele and the other for variants [10]. An internal positive control (IPC), *GAPDH*, was added to all of the wells in order to verify successful amplification.

We selected the target *TPMT* single-nucleotide polymorphisms (SNPs) based on thiopurine dosing guidelines [11] and the genotype frequencies that have been reported in Korean populations and elsewhere [12, 13]. After designing several sets of primers using Primer3 (http://primer3.sourceforge.net/), we selected the most efficient primer pairs with the smallest and largest cycle thresholds (Cts) between the wild-type and variant signals. The primer and probe sequences for the real-time PCR system are proprietary information of the manufacturer (Bioneer, Daejeon, South Korea). The real-time PCR reactions were carried out on an Exicycler real-time system (Bioneer).

Each 50 *μ*L reaction mixture included 5 *μ*L of template DNA, *TPMT*-specific primers, dual-labeled fluorogenic 5′ nuclease *TPMT*-specific probes (5′-FAM; 3′-Dabsyl), a dual-labeled fluorogenic 5′ nuclease *GAPDH*-specific IPC probe (5′-TAMRA; 3′-BHQ1), DNA polymerase, dNTPs, and a stabilizer. The amplification protocol included an initial denaturation at 95°C for 10 minutes, followed by 45 cycles of denaturation at 95°C for 20 seconds and annealing and extension at 55°C for 30 seconds. The results were considered successful if the IPC was amplified with a Ct < 30.

To determine the required minimal DNA concentration, various concentrations of DNA samples (100 ng/*μ*L, 30 ng/*μ*L, 10 ng/*μ*L, 5 ng/*μ*L, and 1 ng/*μ*L) were isolated from 5 patients for 3 consecutive days, tested in quadruplicate and compared. To determine the analytical limit of detection, serially diluted variant templates (1 × 10^7^–1 × 10^2^ copies/*μ*L) were tested. An automatic interpretative software program for real-time PCR assays, which was based on Ct differences of 2, was used as previously reported [10].

### 2.2. Validation of the Allele-Specific Real-Time PCR System

To validate the accuracy of the allele-specific real-time PCR genotyping method, we performed both PCR-direct sequencing and allele-specific real-time PCR using DNA samples from 244 patients who were treated with azathioprine or mercaptopurine. Genomic DNA was extracted from 2 mL of ethylenediaminetetraacetic-acid-(EDTA-) anticoagulated blood using a QIAamp DNA mini kit (Qiagen, Valencia, CA, USA) in accordance with the manufacturer's instructions. In addition, a commercially available *3B genomic DNA (NA09301) sample and *3C genomic DNA (NA03579) sample were also analyzed (Coriell Institute, Camden, NJ, USA). Bidirectional sequencing was conventionally performed on an ABI Prism 3130XL Genetic Analyzer (Applied Biosystems, Foster City, CA, USA). Details regarding the primers used in the sequencing analysis are available upon request.

## 3. Results

### 3.1. Development and Establishment of the Allele-Specific Real-Time PCR System

We selected three major target SNPs: *TPMT* c.238G > C (*2); c.460G > A (*3A and *3B); c.719A > G (*3A and *3C). All IPCs were perfectly amplified. We found that all of the target SNPs were successfully amplified using the specifically designed allele-specific primers and the 5′ nuclease probe. At least 5 ng/*μ*L of the template DNA was needed to yield reproducible results. The analytical limit of detection was 1 × 10^2^ copies/reaction for 238G > C and c.460G > A and was 1 × 10^3^ copies/reaction for c.719A > G. A concentration of 1.0 ng/*μ*L corresponded to 1 × 10^3^ copies/*μ*L.

**Figure 1:**
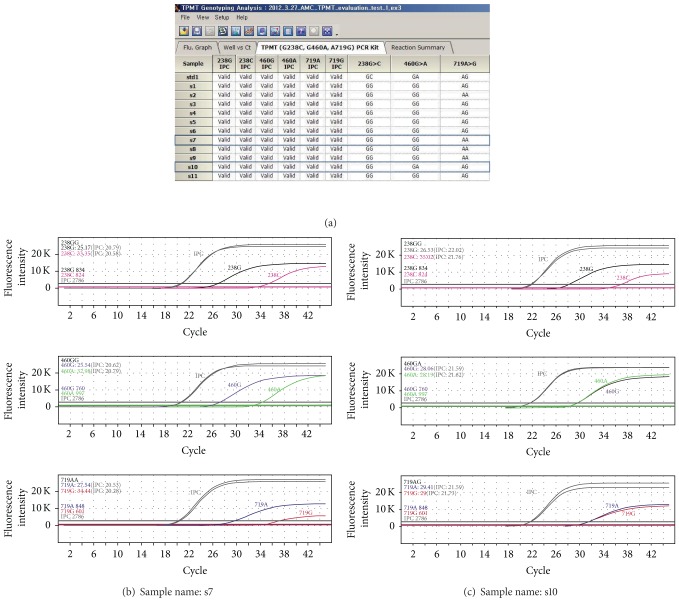
Examples of the automatically interpreted results. Typical displays are shown. (a) Each row shows all of the interpreted results for the three SNPs of one sample. (b) Simultaneous display of all of the reaction curves for the three SNPs of one wild-type sample. (c) Simultaneous display of all reaction curves for the three SNPs of one sample (460G > A and 719A > G heterozygotes).

All of the interpreted results of each specimen are summarized in each row, and the results from all of tested samples are displayed in Figure 1(a). In addition, all of the reaction curves generated for the 3 SNPs from the selected samples are simultaneously displayed (Figures 1(b) and 1(c)), allowing easy and convenient visualization of the reaction curves.

### 3.2. Validation of the Allele-Specific Real-Time PCR System

In all 246 DNA samples (244 patients and 2 commercially available samples), all of the homozygotes demonstrated ΔCt values >8.0, whereas all of the heterozygotes demonstrated ΔCt values <1.0. No ambiguous results were obtained, and all of the data are in agreement with those obtained using the PCR-direct sequencing method. The allele frequency of TPMT *3C was 2.05% (10 of 488 alleles). All of the patients with variant alleles were heterozygotes, and no homozygotes were detected. No TPMT *2, *3A, or *3B alleles were observed in this Korean population.

## 4. Discussion

We developed and validated an allele-specific real-time PCR system (Bioneer) that uses an automatic interpretation function for thiopurine dose genotyping. It was used to assess several major SNPs: TPMT *2, *3A, *3B, and *3C. Based on the thiopurine dosing guidelines [11] and population genotype data [12, 13], we were able to consider the genotyping of these 3 SNPs as sufficient for thiopurine dose adjustment in our population. Although the allele frequency of *6 (c.539A > T and p.Tyr180Phe) has been reported as 1.27% (3 of 256) [14] and 0.25% (2 of 800) in the Korean population [13], *6 is not currently a clinically relevant SNP in the Clinical Pharmacogenetics Implementation Consortium guidelines [11]. Teml et al. [15] recommended using a genotyping strategy to replace the measurement of TPMT activity only if either a complete genetic analysis of all of the currently known functionally relevant TPMT alleles is conducted or if the selection of all of the frequently known alleles in a certain ethnic population is performed. The platform for TPMT *6 could be included if data on Korean patients are sufficiently accumulated for the modification of thiopurine dosing guidelines.

To validate our method, we assayed DNA samples from 244 patients and two commercially available genomic DNA samples, the genotypes of which had previously been confirmed using the PCR-direct sequencing method. We found that the results of the two methods were in excellent agreement. Because the SNPs of *TPMT *are variations at the germline level, they theoretically appear with frequencies of 0%, 50%, and 100%. A Ct difference ≥2 between a variant and the wild-type allele indicates a ≥4-fold difference between the amount of wild-type and variant genomic DNA that are present [10]. Therefore, we considered a Ct difference of 2 to be sufficient for distinguishing between wild-type and variant alleles. We actually found that the Ct differences were ≥8 for all of the homozygotes and <1 for all of the heterozygotes. Hence, none of our findings were inconclusive. We could not validate TPMT *2 because of difficulties obtaining adequate samples, and this is a limitation of this study. However, because TPMT *2 has been found to be absent in the Korean population [13, 14], this system could be suitable for *TPMT* genotyping of Koreans. 

Although real-time PCR requires less time and effort than conventional sequencing, laboratory personnel with little experience may have difficulty interpreting the results. We thus devised an automatic algorithm for the interpretation of real-time PCR results based on the Ct differences [10]. This software can reduce interpretation errors and is timeefficient. In addition, the automatically interpreted results can be connected to a laboratory information system to automatically generate pharmacogenetic interpretation reports, thus saving time and effort in the preparation of laboratory reports. In addition, because our genotyping system is a ready-to-use kit, the time needed for reagent preparation is negligible. In terms of cost, the sequencing method costs $50 per exon, so $150 for three exons per specimen. In contrast, our allele-specific real-time PCR method is expected to cost $50 per specimen; thus, one-third of the cost of the sequencing method.

In conclusion, the system described here allows accurate and timely genotyping in a clinical laboratory setting, allowing the adjustment of the thiopurine dose based on the genotyping results of each patient.
